# The Cambridge Mindreading Face-Voice Battery for Children (CAM-C): complex emotion recognition in children with and without autism spectrum conditions

**DOI:** 10.1186/s13229-015-0018-z

**Published:** 2015-04-23

**Authors:** Ofer Golan, Yana Sinai-Gavrilov, Simon Baron-Cohen

**Affiliations:** Department of Psychology, Bar-Ilan University, Ramat-Gan, 5290002 Israel; Autism Research Centre, Department of Psychiatry, Cambridge University, Douglas House, 18b Trumpington Road, Cambridge, CB2 2AH UK; Cambridgeshire and Peterborough NHS Foundation Trust, CLASS Clinic, Fulbourn Hospital, Cambridge, CB21 5EF UK

**Keywords:** Emotion recognition, Complex emotions, Facial expressions, Prosody, Theory of mind, Empathy, Autism spectrum conditions

## Abstract

**Background:**

Difficulties in recognizing emotions and mental states are central characteristics of autism spectrum conditions (ASC). However, emotion recognition (ER) studies have focused mostly on recognition of the six ‘basic’ emotions, usually using still pictures of faces.

**Methods:**

This study describes a new battery of tasks for testing recognition of nine complex emotions and mental states from video clips of faces and from voice recordings taken from the *Mindreading* DVD. This battery (the Cambridge Mindreading Face-Voice Battery for Children or CAM-C) was given to 30 high-functioning children with ASC, aged 8 to 11, and to 25 matched controls.

**Results:**

The ASC group scored significantly lower than controls on complex ER from faces and voices. In particular, participants with ASC had difficulty with six out of nine complex emotions. Age was positively correlated with all task scores, and verbal IQ was correlated with scores in the voice task. CAM-C scores were negatively correlated with parent-reported level of autism spectrum symptoms.

**Conclusions:**

Children with ASC show deficits in recognition of complex emotions and mental states from both facial and vocal expressions. The CAM-C may be a useful test for endophenotypic studies of ASC and is one of the first to use dynamic stimuli as an assay to reveal the ER profile in ASC. It complements the adult version of the CAM Face-Voice Battery, thus providing opportunities for developmental assessment of social cognition in autism.

## Background

The ability to understand other people’s emotional and other mental states underlies social skills and is a key process in the development of empathy [1]. The ability to discriminate emotions starts during the first year of life. Infants as young as 10 weeks of age respond differentially to their carer’s emotional states, expressed in both the face and voice [2]. By 7 months, infants detect incongruence between facial and vocal expressions of emotions [3]. During their second and third years of life, children start using mental state words in their speech [4]. Throughout childhood, the accuracy and speed of emotion recognition (ER) improve [5], children’s emotional vocabulary expands, and they are able to recognize more subtle mental states [6]. Emotion and mental state recognition skills continue to develop into adolescence and adulthood.

Emotion and mental state recognition are core difficulties in autism spectrum conditions (ASC) [7-9]. Most ER studies carried out with individuals with ASC have focused on the recognition of six emotions (happiness, sadness, fear, anger, surprise and disgust). These so-called ‘basic’ emotions are expressed and recognized cross-culturally [10] and are to some extent neurologically distinct [11], though it should be noted that the number of emotions that are recognized cross-culturally may exceed six [12]. In ASC, some studies report difficulties in recognition of basic emotions [13-16]. Other studies, however, have found no difficulties in recognition of the basic emotions in children with ASC [17-20]. In contrast, studies investigating recognition of *complex* emotions and other mental states by children with ASC have shown more conclusive results. Generally, complex emotions involve attributing a cognitive state as well as an emotion and are more context and culture dependent [11]. They may be belief- rather than situation-based emotions [21], for example, *disappointed*. They may also be self-conscious emotions, for example, *proud* or *embarrassed* [22]. Typically developing children start recognizing and verbally labelling complex emotions like embarrassment, pride and jealousy by the age of 7 [21,23]. Studies report deficits in complex ER in individuals with ASC on various tasks, including ER from pictures of the eyes [24], from facial expressions [25], from linguistic contextual cues [26,27] and from holistic, multimodal scenes [28,29]. These studies suggest that children with ASC, although initially delayed in the development of *basic* ER skills, may achieve this developmental milestone during their school years or successfully compensate for their basic ER difficulties through explicit cognitive, language-based or perceptual mechanisms [30]. An assessment of ER difficulties in children with ASC therefore needs to address more complex mental states. The current study focuses on recognition of complex emotions to fill a gap in the existing literature and to provide a new test of complex ER using dynamic stimuli.

Among adults with ASC, there is growing evidence for difficulties in the recognition of complex emotions or subtle versions of basic emotions [31-34]. However, as mentioned above, there are not many complex ER tasks available for children. Existing tasks have mostly used still pictures [24]. Those that included faces in motion [28] have tended to include only a narrow range of complex emotions. As far as we are aware, there has not yet been any study of children testing complex ER in voices alone. Therefore, there is a need for a test that assesses ER in a variety of complex emotions, in both visual and auditory channels, using motion in the visual task, to get closer to the demands of the real world, while using validated stimuli that are standardized and therefore useful for research and clinical purposes.

In this study, we present such a battery: ‘The Cambridge Mindreading Face-Voice Battery for Children’ (or the CAM-C). This is an adaptation of a complex ER battery for adults [34]. The CAM-C includes nine different complex emotions. The battery provides ER scores for faces and for voices, as well as for the number of emotions correctly recognized. The objectives of the current study were twofold: (a) to compare ER abilities of children with ASC and typically developing controls and (b) to examine the psychometric properties of the CAM-C battery, in terms of reliability, concurrent validity and ability to differentiate between children with ASC and typically developing children in ER skills.

Using this battery, we assessed differences between 8- and 11-year-old children with high-functioning ASC and a typically developing matched control group. We predicted that the ASC group would have lower scores on the battery tasks compared to controls. In addition, we predicted that CAM-C scores would correlate negatively with the level of autistic symptoms [24,29,35] and positively with age [36] and with IQ [37,38]. Correlations with the child version of the ‘Reading the Mind in the Eyes’ (RME) [39], an existing complex ER task, were also calculated to examine the CAM-C battery’s concurrent validity.

## Methods

### Participants

The research was approved by the Cambridge University Psychology Research Ethics Committee. Participation required informed consent from parents and verbal assent from children. The ASC group comprised 30 children (29 boys and 1 girl), aged 8.2 to 11.8 (*M* = 9.7, SD = 1.2). Participants had all been diagnosed with ASC by a psychiatrist or clinical psychologist in specialist centres using established criteria [40,41]. They were recruited from a volunteer database (at www.autismresearchcentre.com) and a local clinic for children with ASC. A control group from the general population was matched to the clinical group. This comprised 25 children (24 boys and 1 girl), aged 8.2 to 12.1 (*M* = 10.0, SD = 1.1). They were recruited from a local primary school. Parents reported their children had no psychiatric diagnoses and special educational needs, and none had a family member diagnosed with ASC. All participants were given the Wechsler Abbreviated Scale of Intelligence (WASI) and scored above 80 on both verbal and performance scales. To exclude ASC, participants’ parents filled in the Childhood Autism Spectrum Test (CAST) [42]. None of the control participants scored above the cut-off point of 15. All but two participants in the ASC group scored above the cut-off. These two participants scored below the cut-off due to several unanswered items. However, since the CAST is a parental report screening questionnaire, the clinical diagnosis received earlier was deemed more valid and these participants were not excluded from the sample. The two groups were matched on sex, age, verbal IQ and performance IQ. The groups’ background data appears in Table 1.

**Table 1:** **Means, SDs and ranges of chronological age, CAST and WASI scores for ASC and control groups**

	**ASC group (** ***n*** **= 30)**		**Control group (** ***n*** **= 25)**		***t*** **(53)**
	**Mean (SD)**	**Range**	**Mean (SD)**	**Range**	
CAST	19.7 (4.3)	11-28	3.4 (1.7)	0-6	18.33**
Age	9.7 (1.2)	8.2-11.8	10.0 (1.1)	8.2-12.1	.95
WASI VIQ	112.9 (12.9)	88-143	114.0 (12.3)	88-138	.32
WASI PIQ	111.0 (15.3)	84-141	112.0 (13.3)	91-134	.27
WASI FIQ	113.5 (11.8)	96-138	114.8 (11.9)	95-140	.39

CAST, Childhood Autism Spectrum Test; WASI, Wechsler Abbreviated Scales of Intelligence. **P < .001. For all the other measures, *P* > .1.

### Instruments

#### The CAM-C: test development

Nine emotional concepts were selected from a developmentally tested emotional taxonomy [23,43]: *amused*, *bothered*, *disappointed*, *embarrassed*, *jealous*, *loving*, *nervous*, *undecided*, and *unfriendly*. The selected concepts included emotions that are developmentally significant, subtle variations of basic emotions that have a mental component and emotions and mental states that are important for everyday social functioning.

For each emotional concept, three face items and three voice items were created using silent video clips of facial expressions and audio clips of short verbalizations spoken in emotional intonation (all 3 to 5 s long). The face and voice clips were taken from an interactive guide to emotions (www.jkp.com/mindreading) [43]. Faces and voices were portrayed by professional actors, both male and female, of different age groups and ethnicities. Three foils were set for each item, using the emotion taxonomy. Selected foils were either the same developmental level or easier levels than the target emotion. Foils for vocal items were selected so they could match the verbal content of the scene but not the intonation (for example, ‘You’ve done it again’, spoken in *amused* intonation, had *interested*, *unsure* and *thinking* as foils). All foils were then reviewed by two independent judges (doctoral students, who specialize in emotion research), who had to agree no foil was too similar to its target emotion. Agreement was initially reached for 91% of the items. Items on which consensus was not reached were altered until full agreement was achieved for all items.

**Figure 1:**
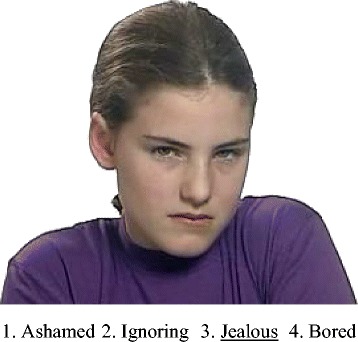
An item example from the face task (showing one frame of the full video clip). Note: Image retrieved from *Mindreading: The Interactive Guide to Emotion*. Courtesy of Jessica Kingsley Ltd.

Two tasks, one for face recognition and one for voice recognition, were created using DMDX experimental software [44]. Each task started with an instruction slide, asking participants to choose the answer that best describes how the person in each clip is feeling. The instructions were followed by two practice items. In the face task, four emotion labels, numbered from 1 to 4, were presented after playing each clip. Items were played in a random order. An example question showing one frame from one of the clips is shown in Figure 1. In the voice task, the four numbered answers were presented before and while each item was played, to prevent working memory overload. This prevented randomizing item order in the voice task. Instead, two versions of the task were created, with reversed order, to avoid an order effect. A handout with definitions for all the emotion words used in the tasks was prepared.

The tasks were then piloted with 16 children - 2 girls and 2 boys from 4 age groups - 8, 9, 10 and 11 years of age. Informed consent was obtained from parents, and verbal assent was given by children prior to participation in the pilot. Children were randomly selected from a local mainstream school and tested there individually. The tasks were played to them on two laptop computers, using headphones for the voice task. To avoid confounding effects due to reading difficulties, the experimenter read the instructions and possible answers to the children and made sure they were familiar with all the words, using the definition handout, where necessary. Participants were then asked to press a number from 1 to 4 to choose their answer. After choosing an answer, the next item was presented. No feedback was given during the task.

Next, item analysis was carried out. Items were included if the target answer was picked by at least half of the participants and if no foil was selected by more than a third of the participants (*P* < .05, binomial test). Items which failed to meet these criteria were matched with new foils and played to a different group of 16 children, until they all met criteria. The final task included 27 items in the face task and 27 in the voice task, representing the nine emotional concepts. In addition, the following measures were used:

#### Childhood Autism Spectrum Test (CAST) [42]

The CAST is a parental questionnaire designed specifically to screen school-age populations for ASC. Scores range from 0 to 31, and the higher the score, the more autism spectrum features the child possesses. In a community sample study [45], the CAST was validated against existing validated diagnostic protocols. With a cut-off score of 15, it discriminated well between children with ASC and typically developing children, with a sensitivity of 100% and specificity of 97%. Its test-retest reliability in a community sample was 0.83 [46].

#### Reading the Mind in the Eyes (RME) - child version [39]

This test of complex mental state recognition consists of 28 photographs of the eye region of the human face, each surrounded by four words. Participants are asked to pick which of the four words best describes what the person in the photo is thinking or feeling. The task is a verbally simplified version of the RME test for adults [24,47]. Children with ASC score significantly lower on this task, compared to matched controls from the general population. Test-retest reliability of the RME, calculated for a subsample of 21 children from the ASC group who took the task twice with a 10- to 15-week time difference, was *r* = .64 (*P* < .01).

#### Wechsler’s Abbreviated Scales of Intelligence (WASI) [48]

This brief measure of intelligence consists of four subtests which provide verbal, performance and full-scale IQ scores. The verbal IQ (VIQ) is comprised of the Vocabulary and Similarities subtests, and the performance IQ (PIQ) includes the Block Design and Matrix Reasoning subtests. These four comprise the Full-Scale IQ (FIQ) and take approximately 30 min to administer. The WASI has been shown to have an internal consistency reliability of .96 and was originally validated against full measures of intelligence for children [48].

### Procedure

Participants with ASC were tested at the Autism Research Centre in Cambridge. Controls were tested at a local school. All participants were tested individually. Prior to undertaking the ER tasks, children completed the WASI, in order to confirm that none had an IQ below 70. The final version of the tasks was presented to the participants on a laptop computer with a 15-in. screen. Headphones were provided for the voice task. The experimenter read the instructions and the questions and answers for all items with the participants, and asked if they were familiar with all the possible answers. If the child was not familiar with a word, it was defined using the definition handout. There was no time limit to answer each item. Completion of the whole battery took about 45 min, including breaks. The RME task was completed during the same session and took about 15 min. Administration order of the three ER tasks (CAM-C face, CAM-C voice and RME) was randomized. Participants’ parents filled in the CAST in advance.

## Results

Facial and vocal scores were calculated as the number of correct answers in each of the tasks. Emotional concepts were counted as correctly recognized if at least four out of the concept’s six items were answered correctly (*P* < .05, binomial test). All participants scored above chance on the face task, and all but one participant from the ASC group scored above chance on the voice task. There were no ceiling effects.

### Between-group findings

In order to check for group and modality differences on complex ER, a multivariate analysis of variance (MANOVA) with repeated measures was conducted, with modality (face, voice) as the within-subject factor and group (ASC, controls) as the between-group factor. The analysis yielded a significant main effect for group (*F*[1,53] = 21.62, *P* < .001, *η*^2^ = .29), with the control group scoring higher than the ASC group. Modality had a significant main effect (*F*[1,53] = 5.17, *P* < .05, *η*^2^ = .09), with participants scoring higher on the voice task. No significant interactions of group and modality were found (*F*[1,53] = .22, n.s.). Univariate analyses of variance for the face and voice tasks showed a lesser performance in the ASC group, compared to the control group, on both tasks. A separate univariate analysis of variance for the number of emotional concepts correctly recognized by participants yielded a significant group effect, with the control group recognizing significantly more emotional concepts than the ASC group. The task scores and the number of emotional concepts correctly recognized by participants in the two groups are presented in Table 2.

**Table 2:** **Group means and standard deviations,** ***F*** **scores, and effect sizes for CAM-C battery**

	**ASC**	**Control**	***F*** **(1,53)**	***η*** **2**
Face task (max = 27)	15.0 (3.9)	19.2 (3.7)	17.1**	.25
Voice task (max = 27)	16.4 (3.6)	20.1 (3.5)	17.6**	.26
Concepts recognized (max = 9)	4.6 (1.7)	6.6 (1.9)	20.22**	.28

**P < .001.

In order to compare the recognition of individual emotional concepts between the two groups, goodness-of-fit tests were performed for the proportions of participants who correctly recognized each concept in the two groups. Table 3 shows proportions of participants of the two groups who recognized each of the nine concepts. As shown in Table 3, compared to the control group, a significantly smaller proportion of individuals in the ASC group correctly recognized *unfriendly*, *disappointed*, *jealous*, *nervous*, *bothered* and *amused*.

**Table 3:** **Proportion of participants who correctly recognized the nine CAM-C concepts**

**Emotional concept**	**ASC**	**Controls**	***χ*** ^**2**^ **(1)**
	**(** ***n*** **= 30)**	**(** ***n*** **= 25)**	
Unfriendly	30%	60%	4.99*
Disappointed	53%	84%	5.83*
Embarrassed	33%	44%	0.66
Jealous	60%	88%	5.39*
Loving	73%	72%	0.01
Nervous	40%	72%	5.63*
Bothered	53%	84%	5.83*
Amused	40%	72%	5.63*
Undecided	73%	84%	0.91

*P < .05.

### Psychometric properties of the CAM-C

Over and above group, participants scored an average of 16.89 (SD = 4.36) on the face task and 18.07 (SD = 3.97) on the voice task and correctly recognized on average 5.49 (SD = 2.08) emotional concepts. As reported above, a significant difference between face and voice task scores was found. However, when participants’ WASI verbal IQ scores were statistically controlled for, this difference became non-significant.

In order to investigate the relation between CAM-C scores and other study measures, correlation analysis was conducted. Due to the relatively small group size, and since there were no differences between correlations in the ASC group and the control group, correlations were only calculated for the two groups combined. The analysis, presented in Table 4, shows the hypothesized negative correlations between CAST scores and CAM-C scores were indeed significant. Age was also positively correlated with CAM-C scores. WASI verbal IQ was positively correlated only with vocal task scores and with the number of emotional concepts correctly recognized. WASI performance IQ was unrelated to any of the tasks. In addition, CAM-C face and voice task scores were positively correlated with each other (*r* = .60, *P* < .001).

**Table 4:** **Correlations of CAM-C scores with background measures and with an external criterion**

	**CAST**	**Age**	**WASI VIQ**	**WASI PIQ**	**RME**
Face task	−.54**	.53**	.21	.04	.35**
Voice task	−.48**	46**	.42**	.00	.40**
Concepts recognized	−.53**	.57**	.35**	.08	.36**

CAST, Childhood Autism Spectrum Test; RME, Reading the Mind in the Eyes - children version; WASI, Wechsler Abbreviated Scale of Intelligence. **P < .01.

Power calculations for the tasks (with *α* = 0.01) show they distinguish well between the ASC and control groups: 1-β = 0.951 for the face task, 0.923 for the voice task and 0.949 for the number of emotional concepts recognized. In order to examine test-retest reliability, 21 children from the ASC group took the CAM-C twice, with 10 to 15 weeks between the two assessments. This was part of an intervention study in which these children served as no-intervention controls. Test-retest correlations were *r* = .74 for the face task and *r* = .76 for the voice task (*P* < .001 for both). Finally, the child version of the RME correlated positively with all CAM-C scores (with the face task: *r* = .35, with the voice task: *r* = .40, with the number of emotional concepts correctly recognized: *r* = .36, *P* < .01 for all). This served as an external criterion and provided support for concurrent validity.

## Discussion

The current study tested if there are differences in complex ER between children with ASC and typically developing children. This was examined using the CAM-C, a new battery, testing complex ER in both facial and vocal expressions. As predicted, the ASC group had more difficulties recognizing complex emotions from faces and voices and recognized fewer emotional concepts, compared to the control group, even when controlling for age and verbal IQ. These results support previous findings of difficulties in complex emotion recognition in children with ASC [25,27-29,49]. The CAM-C battery demonstrated good test-retest reliability and concurrent validity. Scores were positively associated with participants’ age and negatively associated with the level of autistic symptomatology.

Children with ASC showed specific difficulties in the recognition of six out of the nine complex emotions and mental states tested: *disappointed*, *jealous*, *nervous*, *unfriendly*, *bothered* and *amused*. The grounds for these difficulties are discussed in reference to two main factors characterizing complex emotions [4,11]: complexity (that is, combining several basic emotions and mental states) and subtlety (that is, toning down an emotional expression or attempting to conceal it).

Typically developing children have been found to understand and recognize complex emotions such as *jealous*, *disappointed* and *embarrassed* between the ages of 7 and 10 [36,50]. Indeed, our findings show that more than 80% of the control group recognized jealousy and disappointment successfully. However, only 60% of the participants in the ASC group recognized the concept *jealous*, which includes restrained hostility towards someone as a result of social comparison [51]. Common errors included mislabelling facial expressions of *jealous* as *disappointed*, possibly because of focusing on the mouth region of the face, which resembles being unhappy. Relying on the mouth area for ER while disregarding the eyes is characteristic of people with ASC [47,52], particularly in complex emotions [31]. Whereas this may sometimes suffice when interpreting basic emotions (for example, *happy* or *sad*), configural cues, as well as theory of mind, are required for recognition of complex emotions like *jealous*. Voice items for the concept of *jealous* were mislabelled as *teasing* (‘I can do better than you’) or *bossy* (‘I deserve that car more than him’), failing to combine linguistic and paralinguistic components of the verbalizations.

Children with ASC also showed difficulties in the recognition of *disappointment*, which involves sadness due to a failed expectation [53]. Only 53% of the participants in the ASC group correctly recognized this emotion, compared to 84% of the controls. Common errors included mislabelling it as *thinking* and *unsure* for faces, possibly due to the gaze being directed downwards, away from the camera. Participants may have failed to integrate this cue with the unhappy mouth cue. *Disappointed* voice items were commonly mislabelled as *ashamed* (‘I should have won’) and *hurt* (‘I tried so hard’). Whereas these labels capture the emotion’s negative valence, they do not elicit the failed expectation from the verbalizations.

Interestingly, no group difference was found for the recognition of *embarrassed*. Though a larger proportion of controls (44%) recognized this emotion, compared to the ASC group (33%), this difference was not significant. Common errors for face items in both groups included *sad* and *jealous*. Voice items were mislabelled as *afraid* (‘Do you think anyone saw me?’) and *wishful* (‘Oh, I wish it hadn’t happened’). Since embarrassment is a complex emotion, dependent on the real (or imagined) presence of others [54], the correct perception of this emotion would be expected to be facilitated by contextual cues, which were not available in the CAM-C. A task employing holistic situations in context [29] may be useful to examine the ER of *embarrassment*.

As noted, participants in the ASC group had significant difficulties with emotional concepts that form more subtle representations of basic emotions. For example, only 53% of children with ASC (compared to 84% of controls) correctly recognized *bothered*, a form of mild anger. Common mistakes included *disbelieving* and *bored* on the face task, and *unsure* (‘What are you doing here?’) and *disbelieving* (‘I wish I didn’t have to do it’) on the voice task. These demonstrate how, when emotional cues are more subtle, children with ASC may miss their presence and interpret them as mental states. Another example for difficulties recognizing subtle expressions can be seen in the example of *nervous*, a mild expression of fear, recognized by only 40% of the ASC group. Common errors were mislabelling a face item as *annoyed* and voice items as *disgusted* (‘Don’t put that near me’), or an emotionally neutral option, such as *asking* (‘How many people are out there?’). These examples show again how in ASC intonation may be disregarded and verbal content may be used to recognize the speaker’s emotion/mental state. An fMRI study of adults with ASC found that the amygdala, a key brain area underlying the detection of fear in others, does not respond differentially to expressions of subtle fear [55].

Interestingly, there was no group difference in the recognition of the positive emotion *loving*. This is consistent with past research showing specific difficulties to others’ negative emotions in children with ASC [56,57]. Nevertheless, the ASC group had difficulties in the recognition of the positive emotion *amused*, a form of reflective joy [58]. Participants with ASC mislabelled it as *interested* or *curious* on the face task, and as *interested* (‘You’ve done it again’) or *excited* (‘Imagine that’) on the voice task, relying on the linguistic cues while missing the paralinguistic cues of the speaker’s smile [59]. These demonstrate that even in the positive emotion domain, as complexity increases, it is harder for children with ASC to integrate the relevant cues, resulting in a misattribution of emotion.

Only 30% of the participants with ASC correctly recognized the concept *unfriendly*. The ASC group mislabelled unfriendly faces as *afraid*, *disgusted* and *shy*. These errors were probably related to the actors moving their faces away from the camera and looking sideways. Failing to recognize a protagonist as unfriendly, as well as mistaking others’ amusement for interest, may be related to the increased risk of teasing and bullying that children with ASC experience [60,61].

Two patterns emerge from the results, which may account for the errors made by participants in the ASC group in complex ER. First, the relative difficulty in interpreting gaze, characteristic of individuals with ASC, may underlie the pattern of results found in the *unfriendly*, *disappointed* and *jealous* face task items. Previous studies have shown that individuals with ASC show diminished performance compared to typically developing controls in inferring mental states from the eyes [24,62] and atypical eye-gaze processing patterns [63,64].

Second, processing of emotion in prosody should be considered in relation to lowered performance of participants with ASC in the voice items. The processing of affective prosody has been found to be impaired among individuals with ASC [65,66], who may show overreliance on verbal information on the account of change patterns in prosodic cues such as pitch and volume that may be more relevant for the recognition of emotion.

The positive correlations of all task scores with age, independent of diagnosis, suggest that ER skills continue to develop in both typically developing children and children with ASC. In addition, as predicted, CAM-C scores were negatively correlated with the participants’ level of autism spectrum symptoms. This finding highlights the ER profile as a potential marker of ASC. Furthermore, since the range of CAST scores was quite narrow in both groups, correlations with the level of autistic traits were potentially lower than they could be if the autism spectrum was more fully represented, for example, by including undiagnosed siblings of children with ASC [67,68].

As predicted, complex emotion voice task scores were positively correlated with verbal ability. This may be related to the need for integration of the stimuli’s verbal content and intonation, which may depend on verbal ability. It may also demonstrate the compensatory reliance on verbal content, employed by individuals with ASC on emotion recognition tasks [30,65], which may be compromised in individuals with poorer verbal abilities. The correlation of verbal ability with the voice task scores may also explain the significant difference between face and voice task scores, over and above group. Indeed, when verbal ability was entered into a MANCOVA as a covariate, the difference between face and voice tasks became non-significant, while the group difference on both tasks remained significant.

Several issues are noteworthy when examining the psychometric properties of the CAM-C. Power calculations for the CAM-C tasks indicated that the battery differentiates well between the two study groups. Test-retest correlations computed for the battery (.74 to .76) suggest that this measure of complex ER is consistent over time. Furthermore, the positive correlations of CAM-C scores with the RME task provide the battery with important measures of external validity. These correlations were significant but moderate (.35 to .40), suggesting they may test different aspects of a common skill. Power levels of the CAM-C show it is sensitive to group differences across all tasks and scores. These data provide support for the CAM-C as a valid and reliable measure of complex ER skills.

### Limitations and directions for future research

Several limitations should be noted. In the current study, validation of participants’ clinical diagnosis in the ASC group was based on the CAST, a screener for ASC that is based on parental report. Future studies should validate participants’ diagnosis on the basis of independent standardized measures, such as the ADOS-2 [69], which could also contribute to the understanding of the association between ASC symptomatology and complex ER deficits. Additional research of the CAM-C is also needed to further investigate its psychometric properties, such as sensitivity and specificity, with a wider age range, a wider range of validation criteria and a larger sample.

Future research may address questions regarding the ability of the CAM-C to differentiate between ASC and other clinical groups, given that it is significantly correlated with the level of autism symptoms. Finally, some studies have examined the question of scan paths in ER using eye tracking [31]. The application of such a paradigm in the study of the CAM-C might further elucidate the mechanisms underlying the profile found among children with ASC in the recognition of complex emotions from dynamic facial stimuli.

## Conclusions

This new battery for testing complex emotion recognition, in the face (using dynamic stimuli) and in the voice, reveals that 8- to 11-year-old children with ASC have difficulties in complex emotion and mental state recognition in both faces and voices. The CAM-C may be useful in intervention research to monitor improvements in this skill or to augment diagnostic assessments [70-72]. It also lends itself to neuroimaging and developmental research in being standardized and validated and may serve as an endophenotypic stimulus set [73]. It will be interesting to apply the CAM-C to other clinical groups in order to establish its sensitivity and specificity to detect strengths and difficulties in ER.

## Abbreviations

ASC: autism spectrum conditions
CAM-C: Cambridge Mindreading Face-Voice Battery for Children
CAST: Childhood Autism Spectrum Test
ER: emotion recognition
RME: Reading the Mind in the Eyes
WASI: Wechsler Abbreviated Scale of Intelligence
